# Universal screening for familial hypercholesterolaemia: how can we maximise benefits and minimise potential harm for children and their families?

**DOI:** 10.1097/MOL.0000000000000952

**Published:** 2024-10-04

**Authors:** Uma Ramaswami, Lorraine Priestley-Barnham, Steve E. Humphries

**Affiliations:** a Lysosomal Disorders Unit, Royal Free Hospital; b Genetics and Genomic Medicine, University College London; c Royal Brompton & Harefield Hospitals, Part of Guy's and St Thomas’ Trust, Harefield Hospital; d Centre for Cardiovascular Genetics, Institute Cardiovascular Science, University College London, London, UK

**Keywords:** maximising-benefit, minimising-harm, proportion variant-positive

## Abstract

**Purpose of review:**

Universal Screening programmes to identify subjects with familial hypercholesterolaemia (FH) have been the subject of much recent interest. However, any screening programme can cause harm as well as having potential benefits. Here we review recent papers using different ages and strategies to identify subjects with FH, and examine to what extent the publications provide quantitative or qualitative evidence of benefit or harm to children and adults.

**Recent findings:**

Three studies have been published over the last 2 years where Universal Screening for FH has been carried out in infancy, at the time of routine vaccinations, or at preschool age. Next-generation sequencing of all known FH-causing genes has been used to determine the proportion of screened individuals, who have total or low-density lipoprotein cholesterol (LDL-C) concentrations above a predetermined threshold (such as >95th percentile), with genetically confirmed FH.

**Summary:**

While we fully support the concept of Universal Screening for FH, which appears feasible and of potential clinical utility at all of the different ages examined, there is little data to document potential benefit or how to mitigate potential harms. Future study protocols should include collection of such data to strengthen the case of roll out of Universal Screening programmes.

## INTRODUCTION

FH is a monogenic autosomal dominant disorder resulting in elevated total and low-density lipoprotein cholesterol (LDL-C) from birth and premature cardiovascular disease (CVD) [1]. An individual who carries a pathogenic variant (also known as a mutation) in any one of four different genes (*LDLR/APOB/PCSK9/APOE*) has heterozygous FH (HeFH), whereas individuals who carry two copies have homozygous FH (HoFH). Rarely, carriers of two variants in a combination of genes can also have the lipid profile of HoFH [2]. Guidelines on the management of FH recommend that an adult individual with FH should be offered intensive lipid-lowering therapy (LLT) to reduce their LDL-C concentration to at least 50% of their baseline value, with European guidelines proposing that LDL-C should be lowered to 1.8 mmol/l, or 1.4 mmol/l in the presence of CVD [3]. Children with a diagnosis of HeFH are offered dietary and lifestyle advice, with LLT usually offered by the age of 8–10 years [4]. The world-wide and UK prevalence of individuals carrying an FH-causing variant is around 1/280 [5^▪^], suggesting there are over 500 000 children and 2 million adults with FH in Europe, of whom probably <10% are currently identified [1].

In most countries, identification of individuals with FH is predominantly achieved by opportunistic case finding of hypercholesterolaemia in primary and secondary care, supplemented by cascade testing of potentially affected family members, of whom, for first degree relatives, on average 50% will also have FH. Cascade testing has been shown to be feasible, acceptable, and cost effective [6] and is widely used in many countries. However, case finding numbers would improve greatly if this were supplemented by a Universal Screening strategy. FH fulfils all the Wilson and Jungner principles for screening, and has the benefit of reaching people across the whole socioeconomic spectrum, and should help to reduce health inequalities, as CVD is the largest cause of premature mortality in deprived areas [7]. Policy documents stress the potential benefits of Universal Screening [8^▪^] and are supported by patient groups in many countries. However, although not widely acknowledged, as was noted by Gray and Austoker in 1998 ‘All screening programmes do harm; some do good as well, and, of these, some do more good than harm at reasonable cost’ [9]. In order to ensure that roll out of Universal Screening for FH does more good than harm at reasonable cost, we first review recent papers on FH Universal Screening, then discuss the different sources of harm and benefit from contrasting screening protocols, and present various approaches where potential harm could be mitigated and benefits maximised. 

**Box 1:**
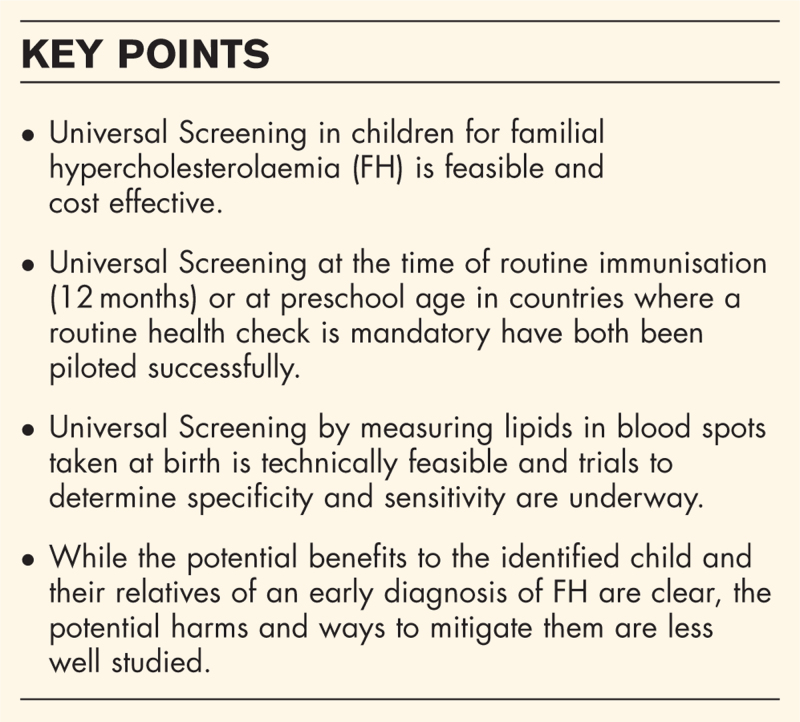
no caption available

## CURRENT FAMILIAL HYPERCHOLESTEROLAEMIA UNIVERSAL SCREENING PROTOCOLS

We carried out a search of published literature over the last 3 years using the terms FH and Universal Screening, including only those where genetic analysis had been performed. As shown in Table 1, comparison with two historical studies from the UK [10] and Slovenia [11^▪▪^] was made with three primary data publications in the past 2 years [12^▪▪^–14^▪▪^]. Two groups reported methods to obtain lipid measures from dried blood spots taken at birth [15,16^▪^] but neither have reported genetic findings.

**Table 1:** Showing two historical US studies in Slovenia and UK (Yellow rows) plus 3 studies published in last 2 years (blue rows)

Study country	Age at screening	Number screened	Threshold for genetic test	Number found M+ (prevalence)	Comment (percentage carrying a mutation)	Reference
UK	12 months (routine MMR vaccination)	10 095	99th %ile TC> 6.0mmol/l	20 (1/500)	Samples from 92 children > threshold sent for genetic test (21.7%). All children had limited FH genetic test^a^ with 17 < threshold carrying a mutation. Overall prevalence 1/273	[10]
Slovenia	Preschool	Unclear	TC >6.0 mmol/l + no family history CVD TC>5.0 mmol/l + family history CVD	76	280 children referred, 170 genotyped (44.7%)^b^	[11^▪▪^]
Australia	12 months (routine MMR vaccination)	488	95th %ile TC>5.3 mmol/l	3 (1/150)	32 had TC >5.3 mol/l and were sent for genetic testing, with 3 carrying a mutation (9.4%)	[12^▪▪^]
Slovenia and Germany	2–6 years	154.658/ 12 133	LDL-C >3.5 mmol/l	219 (1/706)^c^ 26 (1/466)^c^	813 sent for genetic testing (26.9%)^c^ 132 sent for genetic testing ((29.7%)^c^	[13^▪▪^]
Japan	9–10 years	15 665	96.3%ile 3.6 mmol/l	41 (1/380)	580 had LDL-C> threshold of whom (after exclusion of secondary causes) 67 sent for genetic testing (61.2%)	[14^▪▪^]

a All children tested for 50 common FH-causing variants in *LDLR/APOB/PCSK9* with those >99 percentile also having NGS.

b Likely to be an overestimate since some of the variants reported as pathogenic and likely to be VUS or benign.

c Data presented here excluding those with VUS.

All identified studies measure plasma total cholesterol (TC) or LDL-C and/or ApoB and set a threshold above which an individual was designated as ‘likely to have FH’ and a sample sent for genetic testing. Next-generation sequencing (NGS) of at least *LDLR, APOB* and *PCSK9* genes was used and any child carrying a designated FH-causing variant was given the diagnosis of definite FH. This genetic information was then used to test the parents, and sometimes other first-degree relatives, to identify further variant-positive individuals.

In the UK primary care study [10], TC and blood for FH genetic analysis was obtained through a heel prick blood sample taken at ∼12 months of age during routine immunisation (for Measles/Mumps/Rubella (MMR)). Of the 10 094 babies tested, 93 were above the 99th percentile, 20 of those were identified as carrying an FH-causing variant. Since all screened babies had a limited FH test, 17 more with TC below the threshold were also found to carry an FH-causing variant. Reverse-cascade testing of parents identified the expected number of variant carriers, who were subsequently offered LLT. In Slovenia, an unspecified number of preschool children had TC measured and those above two thresholds (Table 1) were referred for genetic testing. This led to the identification of 76 variant-positive children, followed by testing of their relatives

In the three recent studies, the number of screened children ranged from 448 to >160 000, with variant detection rates in those sent for genetic analysis ranging from 9.4% to 61.2%, depending on the stringency of triage of the above threshold children. The overall prevalence of genetically confirmed FH was 1/150 in the Australian study but considerably lower than the predicted 1/280 in the other studies (1/380 to 1/700), as expected if a significant proportion of variant carriers actually have LDL-C below the threshold used.

## MINIMISING HARM AND MAXIMISING BENEFIT

**FIGURE 1:**
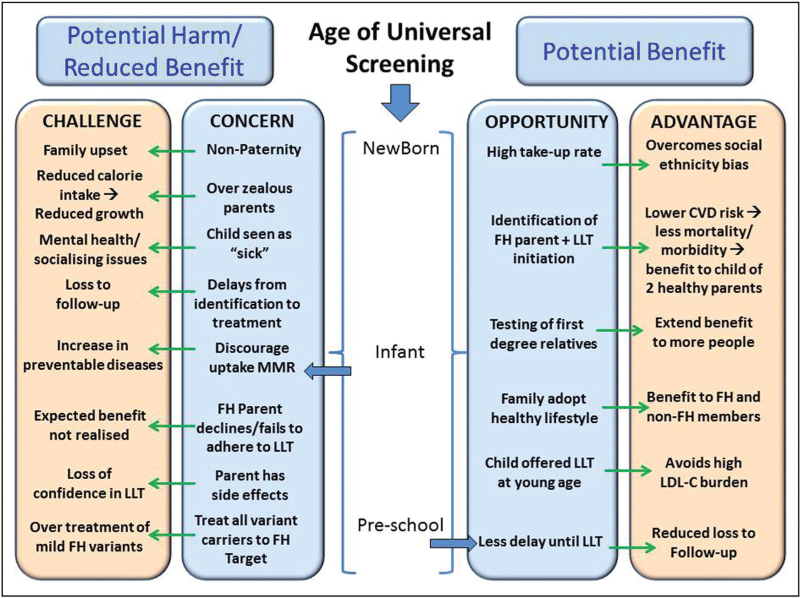
Showing potential benefits and potential harms or reduced benefit associated with Universal Screening for familial hypercholesterolaemia at different times in infancy and childhood.

As shown in Fig. 1 there are a number of clear potential benefits to a Universal Screening programme, whether it is carried out at birth, in infants or at preschool age. Children are not routinely offered LLT until the age of 8–10 years, therefore the primary benefit to the child is the adoption of a healthy lifestyle, extending to other members of the child's family so parents and siblings may also benefit. Cascade testing of first-degree relatives allows the identification of not only the FH parent and siblings but extended family members, though this is highly dependent on the availability and access to genetic testing services locally.

The primary benefit to the child is that their genetic diagnosis is used to identify their affected parent allowing for timely initiation of LLT, thereby reducing their future risk of cardiovascular disease morbidity and mortality.

This benefit is directly underpinned by evidence that children in a family where one parent has suffered a premature death have poorer mental health and educational achievement [17,18]. Where an FH child has been identified at a young age, LLT can be considered by the age of 10 years, meaning their accumulating LDL-C Burden can be prevented [19] protecting their future cardiovascular health. A further potential benefit of Universal Screening at the age of 5–6 years is that there will be less delay before LLT is considered with less chance of the child being lost to follow-up, and therefore denied the potential benefit of the screening programme.

Concerns regarding potential harm or to less than optimum benefit are more complex. Testing the parents of a child identified with an FH-causing variant, and finding that neither parent carries the variant, would raise the possibility of nonpaternity or more remote possibilities such as baby mix-up at birth, both of which are likely to cause upset within the family. Reported nonpaternity rates are surprisingly high, with a review of published data reporting rates from 0.8% to 30% (median 3.7%) [20]. Neither parent carrying the variant found in the child could be explained as a *de novo* variant. We are aware of only one such report of *de novo* variant in an FH family with biological parentage confirmed using genetic markers [21]. Regardless, every Universal Screening programme should have a standard operation procedure to deal with this issue should it arise.

Guidelines for the management of children with FH emphasise the utility of adapting healthy behaviours, but it is possible that some parents may incorrectly perceive this to mean that both fat and calorie intake should be restricted, leading to reduced growth and development. If the child is seen as ‘sick’, and prevented from for example social situations where high-fat food is offered (such as at a friend's birthday party) this may lead to mental health issues and isolation. One report from Norway documents a roughly twofold higher prevalence of eating disorders in individuals with FH than the general population [22^▪^] reinforcing the importance of appropriate dietary counselling for FH given by an experienced healthcare professional.

While modelling has shown that Universal Screening at any age is likely to be highly cost-effective [23,24], this is based on a number of assumptions about a high rate of the uptake and adherence to LLT. It is expected that every parent with a Universal Screening-identified child with HeFH will agree to having a genetic test, and if positive will initiate and adhere to lifelong LLT. However, it is certain that a proportion of such individuals may develop adverse effects and stop LLT because of this or other reasons, such as inability to pay for medication. In such cases, the long term benefit predicted from Universal Screening, and used in cost-effectiveness modelling, will be less than expected. Often when a child is identified by Universal Screening as carrying an FH-causing variant, there may be no strong family history of hyperlipidaemia or of premature CVD and uptake of genetic testing may be low [25]. By contrast, when cascade testing is carried out from an index case having a clinical diagnosis of FH, including significantly elevated LDL-C plus a personal and/or family history of premature CVD, the tested relatives know they are from a family with high CVD risk. As such they may be more willing to be tested, and more interested in taking LLT if found to be variant positive. If adverse effects to LLT occur, there may be a loss of confidence in the use of such medication, leading to the parent being less enthusiastic about their child taking LLT. Ensuring FH is managed by an experienced FH HCP will minimise this risk through appropriate management and education. Finally, when a child has been identified to be FH positive as a baby or infant, there must be a robust mechanism in place for ensuring follow-up for at least 7–8 years, when a repeat lipid measurement would be mandatory in deciding on initiation of LLT. We are unaware of any report of extended cascade testing of family members or follow-up of the children identified in the Slovenia or UK by Universal Screening programmes [10,11^▪▪^], and for both of these, the children would now be over 10 years of age so should have been considered for LLT.

One other potential area of harm is that for all the screening programmes examined, informed consent for lipid and DNA testing of the baby, infant or child was of course obtained from the parent, who had been provided with information about FH and time to consider to consent or not. The child therefore has not had any say as to whether or not they would choose to be screened. An unknown proportion may, when reaching maturity, regret having been tested, and further work on this needs to be carried out. There are different considerations in the setting of newborn screening, when usually only genetic diseases of immediate impact on the health of the baby are examined, but with little information or time given to consider consent. It is currently unclear how best to provide parents with the information they would need to make an informed decision to consent for the additional testing of a newborn baby for an adult onset disease like FH.

## LIMITATIONS OF UNIVERSAL SCREENING PROGRAMMES

For FH Universal Screening programmes based on having a genetic test in those with a high initial lipid measurement, a significant proportion of FH-variant-carrying individuals will be missed. Children with monogenic FH do not all have TC above the threshold for further investigations, and for example in the UK pilot study [10], 46% of children with monogenic FH had TC below the 99th centile. While monogenic FH children who have TC below the 95^th^ centile would be expected to have a lower future risk of CVD than those above the cut-off, their risk is still high. It has been reported that CVD risk is at least twofold higher in monogenic FH than variant negative individuals irrespective of their absolute concentration of LDL-C [26]. Additionally, the LDL-C concentration in some of the variant negative children may increase as they get older. These families will not have follow-up and, even if they are told that FH has not been fully excluded, they may interpret the result this way.

A second issue is the carriers of the *APOB* p.(R2527Q) variant that will be identified by any screening programme. In the UK, in adults with clinical FH where an FH-causing variant can be detected, around 5% carry this *APOB* variant [27] while in the UK pilot study, 40% of the monogenic FH group were identified with this variant [10]. This high prevalence is not unexpected, and from publicly available databases [1], it can be estimated that the frequency of this *APOB* variant in the UK is approximately 1/800 However, patients with this variant typically present with a milder phenotype than those with *LDLR* variants, both in terms of blood concentrations of LDL-C [27] and of future risk of CVD [28]. Therefore, it is likely that any screening programme based on an initial lipid measurement will identify some individuals with a relatively low future risk of CVD who may then be inappropriately treated with intensive LLT to achieve guideline target LDL-C concentrations designed for those with *LDLR*-FH [3]. Currently there is insufficient data in this cohort of children of the benefit of LLT or of future CVD risk, and for children carrying this *APOB* variant, a family history of early onset CVD, including in female relatives, should be taken into account when LLT is considered.

A third limitation is the identification of children carrying a Variant of Uncertain Significance (VUS). All genetic testing can identify a VUS, and rigorous and FH-specific criteria have been developed to guide laboratories about this [29]. In the UK diagnostic laboratories over the last few years, from 19 124 index cases tested, 3–5% had a VUS [30]. The laboratory issues a report stating that a VUS has been identified, and the diagnosis of FH cannot be confirmed without additional data. If such data becomes available, the laboratory re-issues the report stating that the variant is now reclassified as likely to be FH-causing, or that it is likely to be benign. Having a clear protocol of how children identified with a VUS will be managed would be helpful to reduce the potential harm of misleading information being given to parents and families.

A fourth concern is that with every FH programme which refers all children with a lipid measure over a threshold for genetic testing, only a small proportion will be found to carry an FH-causing variant. In the studies shown in Table 1 this ranged from 10% to 61%. The UK study proposed to use the 95th percentile as the threshold for genetic testing in future Universal Screening. For 1000 children screened, this translates into 50 children referred for genetic testing of whom a maximum of 3–4 would be likely to carry an FH-causing variant for a detection rate of 8%. While the care-pathway for the genetically confirmed FH children is usually well mapped out, there is much less clarity about the remaining children who have LDL-C above the screening threshold. We believe that the potential high CVD risk of these children should not be discounted simply because they do not carry a monogenic cause of FH. While some of these children may have elevated TC concentration due to environmental factors (e.g. diet), most are likely to have inherited a greater-than-average number of common LDL-C raising single nucleotide polymorphisms (SNPs). This polygenic aetiology was demonstrated by Talmud *et al.*[31] and subsequently validated [32]. Data from several longitudinal studies have shown that high cholesterol concentrations in childhood persist into adulthood, increasing the risk of premature CVD. For example, a recent paper [33] examined whether childhood risk factors among 38 589 participants, were associated with CVD in adulthood. The risk of CVD for those with a cholesterol concentration in the top 13% was more than twice as high as for those with a cholesterol level in the bottom 20%. We propose that children identified with TC >95th centile, undergo clinical assessment from a paediatric healthcare professional to exclude secondary causes of hyperlipidaemia and to provide healthy lifestyle advice. A single recall at the age of 8 years is recommended to re-assess the lipid profile and reinforce lifestyle and dietary advice. For those children with persistently high LDL-C, LLT should be considered after evaluating additional CVD risk factors.

## CONCLUSION AND FUTURE RESEARCH

Whilst we fully support the aims of Universal Screening for FH, we believe that there should be agreement about measures to be put in place to run alongside the programmes to enhance potential benefits and mitigate potential harms. In particular, identifying 5% of the tested children as having ‘high’ cholesterol but then subsequently not offering any systematic follow-up to around 90% of these children is a potential source of harm. This could be mitigated by follow-up to reduce future CVD risk by offering dietary and lifestyle advice as well as LLT as appropriate. While patient groups are reported to be in favour of FH Universal Screening in newborns [34,35], as pointed out [9], following identification through a screening programme ‘we have to be cautious and distinguish between patient gratitude on the one hand and objective evidence of reduced mortality on the other’. Research should be carried out to follow up identified children at least until they have reached teenage years, to document what proportion will be offered LLT by the age of 10 years and adhere to the prescribed medication and what proportion of children are lost to follow-up, fail to attend clinic visits, or decline treatment. Since there will be additional costs from seeing all variant negative children with TC >95th centile for one baseline clinical assessment and one follow-up visit a full economic analysis would be beneficial. Follow-up of the variant negative children with LDL-C >95th centile should be undertaken, monitoring unwanted effects on anxiety, false reassurance, or control of lipid concentration, which a previous study suggests are likely to be modest [36]. Whilst our proposal requires commissioning of additional resources, we believe tailored monitoring throughout childhood should be mandatory once a modifiable coronary risk factor has been identified which could predispose any child to an enhanced risk of premature CVD in adulthood.

## Acknowledgements

*Ethics approval and consent to participate: Not applicable.*

*Consent for publication: Agreed by all authors.*

*Availability of data and materials: Not applicable.*

*Funding: No honorarium, grant, or other form of payment was given to anyone to produce the manuscript. SEH is an Emeritus BHF Professor and acknowledges funding from the BHF (PG08/008), and the National Institute for Health Research and University College London Hospitals Biomedical Research Centre. All authors acknowledge funding from the National Institute for Health and Care Research Health Technology Assessment (NIHR HTA) (Award ID NIHR134993). The views expressed are those of the authors and not those of the NIHR or the Department of Health and Social Care. Project information available at* *https://fundingawards.nihr.ac.uk/award/NIHR134993*

*Author contributions: S.E.H. wrote the first draft of the manuscript. U.R. and L.P.B. commented on the draft of the manuscript.*

### Financial support and sponsorship

*None.*

### Conflicts of interest

*S.E.H. is the Medical Director of a UCL Spin-off company that offers genetic testing for cardiovascular risk including for FH. U.R. and L.P.B. have no conflicts of interest to declare.*

## REFERENCES AND RECOMMENDED READING

Papers of particular interest, published within the annual period of review, have been highlighted as:

▪ of special interest
▪▪ of outstanding interest
